# Hapten Synthesis and Monoclonal Antibody Preparation for Simultaneous Detection of Albendazole and Its Metabolites in Animal-Origin Food

**DOI:** 10.3390/foods10123106

**Published:** 2021-12-14

**Authors:** Shibei Shao, Xuping Zhou, Leina Dou, Yuchen Bai, Jiafei Mi, Wenbo Yu, Suxia Zhang, Zhanhui Wang, Kai Wen

**Affiliations:** Beijing Laboratory for Food Quality and Safety, Beijing Key Laboratory of Detection Technology for Animal-Derived Food Safety, College of Veterinary Medicine, China Agricultural University, Beijing 100193, China; shaoshibeil@cau.edu.cn (S.S.); 20098297@bua.edu.cn (X.Z.); b20193050410@cau.edu.cn (L.D.); BS20193050465@cau.edu.cn (Y.B.); mijiafei@cau.edu.cn (J.M.); yuwenbo@cau.edu.cn (W.Y.); suxia@cau.edu.cn (S.Z.); wangzhanhui@cau.edu.cn (Z.W.)

**Keywords:** albendazole, metabolites, hapten design, antibody, immunoassay, computational chemistry

## Abstract

Albendazole (ABZ) is one of the benzimidazole anthelmintics, and the overuse of ABZ in breeding industry can lead to drug resistance and a variety of toxic effects in humans. Since the residue markers of ABZ are the sum of ABZ and three metabolites (collectively referred to as ABZs), albendazole-sulfone (ABZSO_2_), albendazole-sulfoxide (ABZSO), and albendazole-2-amino-sulfone (ABZNH_2_SO_2_), an antibody able to simultaneously recognize ABZs with high affinity is in urgent need to develop immunoassay for screening purpose. In this work, an unreported hapten, 5-(propylthio)-1H-benzo[d]imidazol-2-amine, was designed and synthesized, which maximally exposed the characteristic sulfanyl group of ABZ to the animal immune system to induce expected antibody. One monoclonal antibody (Mab) that can simultaneously detect ABZs was obtained with IC_50_ values of 0.20, 0.26, 0.77, and 10.5 μg/L for ABZ, ABZSO_2_, ABZSO, and ABZNH_2_SO_2_ in ic-ELISA under optimized conditions respectively, which has been never achieved in previous reports. For insight into the recognition profiles of the Mab, we used computational chemistry method to parameterize cross-reactive molecules in aspects of conformation, electrostatic fields, and hydrophobicity, revealing that the hydrophobicity and conformation of characteristic group of molecules might be the key factors that together influence antibody recognition with analytes. Furthermore, the practicability of the developed ic-ELISA was verified by detecting ABZs in spiked milk, beef, and liver samples with recoveries of 60% to 108.8% and coefficient of variation (CV) of 1.0% to 15.9%.

## 1. Introduction

**Figure 1:**
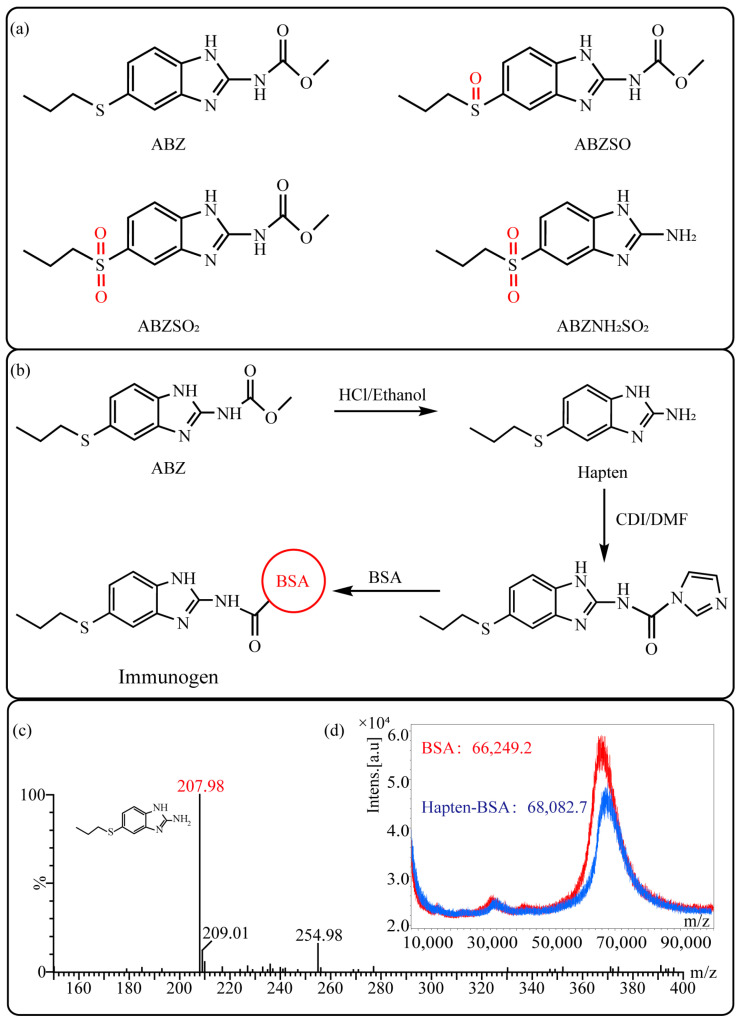
Chemical structures and synthesis route used in the study: (**a**) Chemical structure of four ABZs. (**b**) Synthesis route of hapten and immunogen. (**c**) Mass spectrometry characterization of hapten and (**d**) Matrix-assisted laser desorption/ionization time-of-flight mass spectrometry characterization of hapten- bovine serum albumin (BSA) conjugates.

Albendazole (ABZ, shown in Figure 1a), one of benzimidazoles, is an effective anthelmintic and often used to control soil-transmitted helminth infection in humans and animals. ABZ is usually the first choice for treatment of parasitic diseases, such as cystic echinococcosis and alveolar echinococcosis [1,2,3], and for eliminating lymphatic filariasis in endemic areas [4,5,6]. After administration of ABZ, the kinds of residues that can be monitored depend on the route of administration, target tissue or the detection time after treatment. At early periods, the most likely residues are albendazole sulfoxide (ABZSO) and albendazole sulfone (ABZSO_2_), while albendazole 2-amino sulfone (ABZNH_2_SO_2_) may be the most persistent residue in tissue at longer withdrawal periods [7,8]. Residues of ABZ in animal food can lead to embryonic toxicity for consumer, as well as teratogenic and mutagenic effects [9,10]. The European Union (EU) has set the maximum residue limits (MRLs) for ABZs (sum of ABZ, ABZSO, ABZSO_2_, and ABZNH_2_SO_2_, Figure 1a) at 100 μg/kg (L) in milk/muscle/fat, 500 μg/kg in kidney, and 1000 μg/kg in liver [11]. The U.S. FDA has set different residue limits in different samples, for example, 200 μg/kg in liver, 50 μg/kg in muscle [12]. The Ministry of Agriculture and Rural Affairs of China has set the MRLs (GB 31650-2019) for ABZs (sum of ABZ, ABZSO, ABZSO_2_, and ABZNH_2_SO_2_) at 100 μg/L in milk, and for ABZNH_2_SO_2_ at 100–5000 μg/kg in edible tissues of food-producing species [13]. To ensure food safety, various instrumental methods have been established for determining ABZs in animal-derived food [14,15,16,17,18,19]. Although these methods are highly sensitive, they rely heavily on expensive instruments and skilled personnel, which often cannot meet the current urgent need for fast screening. Thus, there is great interest in the development of accurate and fast methods.

Immunoassays, based on antibody-antigen recognition, offer a convenient and rapid alternative to instrumental methods and are currently the most widely used screening methods in food safety. Though immunoassay has many advantages including high-throughput, rapidity, and on-line detection, few studies have been conducted on immunoassay development due to the unavailability of antibody with high affinity and specificity to all ABZs, especially ABZNH_2_SO_2_. There have been only several reports of antibodies production for benzimidazoles including some of ABZs (Table 1), but all of the antibodies were of limited affinity to ABZNH_2_SO_2_, and the non-specificity binding to various benzimidazoles greatly constrained its application in detection of ABZs. Hapten design is the key to producing antibody with desired affinity and specificity. In previous studies, the strategy of maintaining the common moiety of benzimidazoles, carbamate group as the epitope [20,21,22,23] was used to obtain a broad-spectrum antibody. To the best of our knowledge, no report describing the production of antibody able to sensitively and specifically recognize all four ABZs has been reported despite of the great demand. In the present study, we described the synthesis of one new hapten, production of a Mab with highly specificity, exploration of antibody recognition mechanism, and development of an indirect competitive ELISA (ic-ELISA) for the determination of ABZ and its metabolites in milk and tissue simultaneously with improved sensitivity.

## 2. Materials and Methods

### 2.1. Reagents and Materials

ABZ, ABZSO, ABZSO_2_, ABZNH_2_SO_2_, fenbendazole, flubendazole, mebendazole, oxibendazole, oxfendazole, triclabendazole, carbendazim, and thiabendazole were purchased from J&K Chemical Technology (Beijing, China). Carbonyldiimidazole and other chemical reagents were supplied by Sinopharm Chemical Reagent (Beijing, China). Ovalbumin (OVA), bovine serum albumin (BSA), hypoxanthine aminopterin thymidine (HAT), complete and incomplete Freund’s adjuvant, poly (ethylene glycol) (PEG) 1500, and fetal bovine serum were acquired from Sigma–Aldrich (St. Louis, MO, USA). Goat anti-mouse IgG (HRP labeled) was purchased from Jackson Immuno Research (West Grove, PA, USA). Cell culture medium (DMEM) was supplied by Thermo Fisher Scientific (Waltham, MA, USA). TMB (3,3′,5,5′-tetramethyl benzidine) substrate solution and hydrogen peroxide (H_2_O_2_) were purchased from Beyotime (Shanghai, China). Distilled water used in this study was obtained from a Milli-Q purification system (Bedford, MA, USA). Microplates for ELISA were acquired from Costar (Cambridge, MA, USA). Flat-bottomed high-binding polystyrene cell culture plates were obtained from Corning Life Sciences (New York, NY, USA). Balb/c mice were supplied by Beijing Vital River Laboratory Animal Technology (Beijing, China). All of the buffers used in the immunoassay have been listed in Supplementary Materials.

### 2.2. Preparation of hapten and conjugates

#### 2.2.1. Synthesis and Identification of Hapten

The hapten synthesis route is shown in Figure 1b and briefly described as follows: 500 mg of ABZ was firstly dissolved in 5.0 mL of ethanol in a round bottom flask with a magnetic stirrer, and then 10 mL of hydrochloric acid was added and heated to 80 °C for 30 min with stirring and monitored by thin-layer chromatography. After reaction, the pH value of the mixture was adjusted to 9.0 by using 2 M of sodium hydroxide solution, and extracted twice with 30 mL of ethyl acetate. The organic phase was combined and dried by using 2.0 g water-free sodium sulfate and the precipitate were removed. Then, 1.5 g of 100–200 mesh silicon was added to the organic phase and then dried for subsequent column chromatography (40 g 200–300 mesh chromatography silicone, eluted by petroleum ether: ethyl acetate 4:1). Finally, the hapten was obtained and vacuum-dried for mass spectrometry confirmation as shown in Figure 1c.

#### 2.2.2. Synthesis and Identification of Immunogen and Coating Antigen

Amino groups of the acquired hapten were conjugated to carrier proteins by using carbonyldiimidazole as coupling reagent. Firstly, 9.2 mg of hapten was dissolved in 1.5 mL of DMF and stirred at 200 rpm for 10 min, then 7.6 mg carbonyldiimidazole was added, and the solution was stirred at room temperature (500 rpm) for 3 h to obtain activated hapten for subsequent coupling with carrier protein. Next, 50 mg of BSA was dissolved in 3.5 mL of 0.1 M of sodium bicarbonate solution, stirred at 200 rpm for 10 min, then cooled down via ice-bath. The previous activated hapten was then dropped into the protein solution at a rate of 1.0 mL/min under 1000 rpm stirring, then mixed at 500 rpm for 24 h. The reaction products were dialyzed for 3 days against phosphate buffer solution (PBS, 0.01 M, pH 7.2) under 4 °C. Finally, the product was centrifuged at 5000 rpm for 6 min to harvest the purified supernatant and stored at −20 °C until use. Immunogen was characterized by matrix-assisted laser desorption/ionization time-of-flight mass specTrometry (MALDI-TOF-MS, see Figure 1c) and the conjugation ratio was calculated as follows:

Conjugation ratio = (M (conjugates) − M (BSA))/M (haptens)(1)

Coating antigen was synthesized using the above-mentioned procedure, except OVA was substituted for BSA.

### 2.3. Production of Monoclonal Antibody

All animal experiments were conducted in strict accordance with Chinese laws and guidelines approved by the animal ethics committee of China agricultural university. Eight Balb/c female mice aged 8 weeks were immunized with immunogen (diluted in PBS to 1.0 mg/mL) at a dose of 100 μg per mouse on an identical schedule. For primary immunization, mice were injected subcutaneously with a fully emulsified mixture of equal volumes complete Freund’s adjuvant and prepared immunogen. For enhancement, mice were immunized with an emulsified mixture of immunogen and incomplete Freund’s adjuvant every 3 weeks. A total of 4 immunizations were administered with the last one given via intraperitoneal injection without the adjuvant. To better monitor the serum titer and specificity by ic-ELISA, serum was collected 7–14 days after each immunization, according to results of a dynamics study of the antibody-mediated immune response [24]. Four days after the last boost immunization, mice spleen cells were separated and fused with PEG 1500 pre-treated sp2/0 myeloma cells to prepare hybridomas according to procedures described previously [25,26,27]. The fused cells were cultured in HAT medium for 7 days and were screened by testing the supernatant using ic-ELISA to determine the binding ability. The positive and highly sensitive hybridomas were obtained after subcloning four times using the limiting dilution method. Finally, the hybridomas were intraperitoneally injected into mice, and the ascites collected from mice were extracted and purified with saturated ammonium sulfate to obtain purified Mabs.

### 2.4. Development and Optimization of ic-ELISA

An ic-ELISA was established under the following optimized assay conditions: microplates were coated with 100 μL of coating antigen dissolved in 0.05 M carbonate buffer and incubated at 37 °C for 2 h. The plates were then washed three times for subsequent blocking. A volume of 150 μL/well of blocking buffer was added and incubated at 37 °C for 1.5 h, after which the buffer was removed. For the competitive step, both 50 μL of competitor and 50 μL of antibody working solution were pipetted into each well and incubated at 37 °C for 30 min. The plates were then washed three times, 100 μL/well of goat-anti-mouse IgG-HRP diluted in PBS (1:5000) was added, and the plates were incubated at 37 °C for 30 min. The plates were washed as above, 100 μL/well of newly prepared substrate solution was added, and the plates were incubated at 37 °C for 15 min. Finally, the chromogenic reaction was terminated with 50 μL/well H_2_SO_4_ (2 M). Optical density (OD) values at 450 nm were measured with a Multiskan FC machine by Thermo Scientific (Shanghai, China). The OD450 values were plotted against the analyte concentration on a logarithmic scale, and the generated sigmoidal curve was mathematically fitted to a four-parameter logistic equation using the OriginPro 8.5 software (OriginLab Corporation, Northampton, MA, USA).

Y = (A – D)/(1 + (X⁄C)^B^) + D (2)

where A = response at high asymptote, B = the slope factor, C = concentration corresponding to 50% specific binding (IC_50_), D = response at low asymptote, and X = the calibration concentration.

Several physicochemical factors were optimized to improve the performance of ic-ELISA, including pH and ionic strength of working solution.

#### 2.4.1. Effect of pH

The effect of varying the pH on the ic-ELISA was tested by dissolving the analytes and Mabs in PBS buffer at a specified pH and adding them to the antigen coated plates in equal volumes (50 µL/well). The pH values of 5.5, 6.5, 7.0, 7.4, and 8.5 were tested in the ic-ELISA incubation step with all other parameters of the assay fixed.

#### 2.4.2. Effect of Ionic Strength

The effect of ionic strength of assay buffer on the ic-ELISA performance was studied using different NaCl concentrations of 0.05, 0.1, 0.15, 02, 0.4, and 0.8 M in 0.01 M of phosphate buffer, respectively. The effects of these salt concentrations were evaluated on the ic-ELISA by comparing the ABZ competition curves measured with each buffer at pH 7.4.

### 2.5. Cross-Reactivities and Computational Chemistry Analysis

The specificity of the developed ELISA was assessed using cross-reactivity (CR) determined under optimal conditions. The CRs of ABZs and other widely used benzimidazole anthelmintics, such as fenbendazole, oxibendazole, mebendazole, flubendazole, oxfendazole, triclabendazole, carbendazim, and thiabendazole, were calculated according to the following equation:

CR = IC_50_ (ABZ, μg/L)/IC_50_ (analytes, μg/L) × 100% (3)

The computational chemistry method, which can provide electrostatic potential and conformational information of the molecule regarding antibody recognition, was employed here to study the CRs of immunoassays and the binding interactions between small molecules and antibody. All 3D structures were built in Gaussian 09 (Gaussian Inc., Wallingford, CT, USA) and then optimized by the density functional theory calculations with the M06-2X density functional and TZVP basis set. Basing on the lowest energy conformations, molecular alignments were materialized by molecular overlay modules in Discovery Studio 2019 (Accelrys Software, Inc., San Diego, CA, USA). The degree of molecular superposition was measured by alignment root-mean-square-deviation (RMSD). The quantitative molecular surface analysis in the Multiwfn software package was then applied together with VMD to analyze molecular electrostatic potential (ESP) and map ESP on van der Waals surface [28,29]. The molecule volume, and total polar surface area (TPSA) were extracted using the Multiwfn 3.7(dev) code. Dipole moment (μ) was extracted from the Gaussian output file. The Log *P* was obtained using ChemDraw (PerkinElmer, Waltham, MA, USA). By analyzing steric and electrostatic contour maps of the region around the molecule with respect to changes in affinity, the structure–activity relationship between the drugs and the Mab was studied.

### 2.6. Sample Preparation

Negative samples (milk, muscle, and liver of bovine) were obtained from the National Reference Laboratory for Veterinary Drug Residues (Beijing, China). Tissue samples (muscle and liver) were spiked with ABZNH_2_SO_2_, and milk was spiked with ABZ/ABZSO/ABZSO_2_/ABZNH_2_SO_2_. Three grams (milliliters) homogenized sample were weighed into a 50-mL centrifuge tube with 1.0 g of sodium sulfate for pretreatment. Two milliliters of 50% NaOH (only tissue sample) and 9.0 mL of ethyl acetate were then added to the above tube. The mixture was vortexed for 5 min and centrifuged at 4000 rpm for 10 min. Next, 4.5 mL of the supernatant was transferred into a new tube and dried at 60 °C under a nitrogen stream. One milliliter of n-hexane and 0.5 mL of acetonitrile were added to the dried tube for cleaning and resuspension and followed by centrifugation at 4000 rpm. The upper n-hexane and commixture was discarded then, and 0.1 mL of the remaining liquid was pipetted into a new centrifuge tube and mixed with 0.9 mL PBS (0.01 M) (for milk samples) or 1.9 mL PBS (for tissue samples) for detection. The limit of detection (LOD) was determined as the 10% inhibition concentration (IC_10_) calculated from calibration curves. All measurements were completed in triplicate.

## 3. Results and Discussion

### 3.1. Hapten Design and Characterization

Hapten structure determines the specificity and affinity of the resulting antibody [30]. To produce the antibody that could recognize ABZs, the characteristic structure of these compounds should be maximumly exposed to immune system. Previous studies have designed hapten by maintaining the carbamate group, which is a common structure in major benzimidazoles, to prepare antibodies with broad-spectrum specificities [20]. Some of the obtained antibodies could detect ABZ with affinity of 0.66 to 1.4 μg/L (Table 1), but showed poor affinity to ABZ metabolites especially ABZNH_2_SO_2_, which are critical residue markers of ABZ. Even worse, the specificities of these antibodies were insufficient because of the recognition of many other benzimidazoles with CRs of 3% to 88%. A similar result was also observed in another study, in which the ABZNH_2_SO_2_ was directly used as hapten to produce antibodies against ABZNH_2_SO_2_ [21].

In this study, we designed a new hapten of ABZs as shown in Figure 1b and Table 1. The feature structure of ABZ, the sulfanyl group, was maximumly exposed to immune system to induce an antibody with specific recognition of ABZs by conjugating with proteins at the carbamate moiety. Besides the increasing in complexity of epitope (often means stronger antigenicity) relative to previous ones, another key point in this design is that the retained sulfanyl group of ABZ could better maintain the characteristic structures of ABZs, which are structurally different from other benzimidazoles. The hapten was identified by High performance liquid chromatography-tandem mass spectrometry (HPLC-MS/MS, as shown in Figure 1c) (Agilent Technologies, Santa Clara, CA, USA) and ^1^H nuclearmagnetic resonance spectrometry (NMR, see Figure S1 in Supplementary Materials) (Bruker, Rheinstetten, Germany), and the molecular ions (*m/z*) of hapten were 207.98, indicating that the hapten was successfully obtained. The hapten-BSA conjugate was then used as the immunogen, while hapten-OVA as the coating antigen. The immunogen was characterized by MALDI-TOF-MS (Bruker, Rheinstetten, Germany). The correct molecular weight of the hapten molecular ion and conjugate were observed (see Figure 1d), demonstrating that the hapten had been conjugated to the carrier protein, and the calculated conjugation ratio of hapten to BSA was 8.8:1.

### 3.2. Development and Optimization of the ic-ELISA

**Figure 2:**
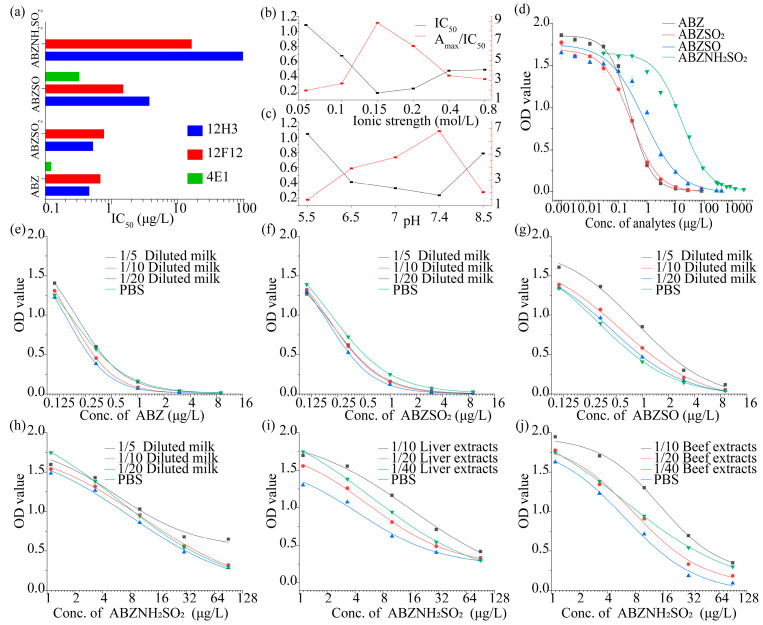
Optimization of ic-ELISA. The IC_50_ was calculated when the A_max_ (Optical density (OD) value of negative control) ranged from 1.0 to 2.0. (**a**) Screening of Mab. Each Mab was estimated using four ABZs separately. The effect of (**b**) pH value and (**c**) ionic strength in the ic-ELISA were evaluated using Mab 12F12 and ABZ. (**d**) Standard curves of ic-ELISA for ABZs. (**e**–**j**) the calibration curves of the ic-ELISA for (**e**) ABZ, (**f**) ABZSO_2_, (**g**) ABZSO, (**h**) ABZNH_2_SO_2_ in phosphate buffer solution (PBS) and milk, and of ABZNH_2_SO_2_ in extracted (**i**) bovine liver and (**j**) beef. Each value represents the average of three independent replicates.

Four ABZs, including ABZ, ABZSO_2_, ABZSO, and ABZNH_2_SO_2_, were used to determine the affinity (expressed by IC_50_) and specificity (expressed by CR) of antibodies using homologous coating antigen. The results indicated that the obtained antibodies 12F12, 12H3, and 4E1 could recognize ABZs with varied affinities (IC_50_ values of antisera were from 0.12 μg/L to beyond 100 μg/L). As shown in Figure 2a, the antibody 4E1 exhibited relatively higher affinity to ABZ and ABZSO, but relatively worse recognition towards ABZNH_2_SO_2_; 12H3 had high affinity to three ABZs but unfavorable affinity to ABZNH_2_SO_2_; while the antibody 12F12 showed high affinity to all four ABZs and was selected for further optimization.

The development and optimization procedure were performed according to previous studies [25,26]. Ionic strength and pH value of working buffer were further optimized. The A_max_ (means the OD value of negative control)/IC_50_ ratio was introduced here to be a criterion for optimization, and a higher ratio value meant a higher sensitivity. It can be seen that the ratio of A_max_/IC_50_ was highest at an ionic strength of 0.15 mol/L (Figure 2b) and pH 7.4 (Figure 2c). Thus, the optimum conditions (pH 7.4 and 0.15 mol/L NaCl in PB buffer, namely, 0.01 M PBS) were used in subsequent experiments.

The sensitivity of the ic-ELISA was characterized by IC_50_ values from standard curves under the optimized conditions, Standard curves of ABZ/ABZSO_2_/ABZSO were established at 27.0, 9.0, 3.0, 1.0, 0.33, 0.11, 0.037, 0.012, 0.004, 0.001, and 0 μg/L in PBS, standard curves of ABZNH_2_SO_2_ were established at 270, 90, 30, 10, 3.33, 1.11, 0.37, 0.12, 0.04, 0.01, and 0 μg/L in PBS. As shown in Figure 2d, the developed ic-ELISA based on antibody 12F12 could detect ABZ, ABZSO_2_, and ABZSO with IC_50_ values below 1.0 μg/L (0.20, 0.26, and 0.77 μg/L), and ABZNH_2_SO_2_ with IC_50_ values of 10.5 μg/L in PBS buffer, which is of the highest affinity so far. The results shown that the sensitivities of the developed ic-ELISA were times better than those of other reported immunoassays for ABZs (Table 1).

### 3.3. Cross-Reactivities and Structure-Activity Relationship Study by Computational Chemistry

In the process of antibody-antigen recognition, cross-reactivity would arise if the configuration of the antigen matches the active pocket of the antibody [31,32]. As shown in Table 2, the ic-ELISA exhibited varied CRs with ABZ (100%), ABZSO_2_ (76.9%), ABZSO (26.0%), ABZNH_2_SO_2_ (1.9%), and other often-used benzimidazoles (fenbendazole, flubendazole, mebendazole, oxibendazole) less than 8%. The relative lower CR value of ABZNH_2_SO_2_ (CR = 1.9%) can easily draw our attention, which shares similar structure with ABZSO_2_ (CR = 76.9%), except for the carbamate group on the right of molecule in the case of ABZSO_2_. As the sulfanyl group of hapten was exposed to immune system as a characteristic epitope in primary hapten design, it would be the site for the antibody recognition. While the remote amide group, which formed after the hapten was coupled to carrier protein, should be masked by carrier protein and contribute much less for antibody recognition. However, the recognition ability of the obtained Mab seemed not in accordance with expectation by analyzing only 2D dimension due to the relative poorer recognition of ABZNH_2_SO_2_ without a methoxyamide or carbamate group. Nevertheless, the benzimidazoles having complex (from a 2D view) side chain groups or phenyl groups (fenbendazole, flubendazole, mebendazole, oxibendazole, Table 2) surprisingly showed higher CRs than ABZNH_2_SO_2_. Though the ic-ELISA was with high sensitivity for detection of all ABZs and far better than previous study, it was appealing to further study on the CR data, and there was considerable interest in understanding at 3D level as well as quantitative analysis. Therefore, computational chemistry analysis was used to provide 3D conformations and quantitative information, such as configuration, electrostatic potential, and hydrophobicity of molecules, to investigate the binding of antibody to compounds. Based on their optimized lowest energy conformations of ABZs and other benzimidazoles that showed cross-reactivities, all molecules were aligned while ABZ was selected as the template. All hydrogen atoms were hidden in the optimized geometric structures for a concise view.

**Figure 3:**
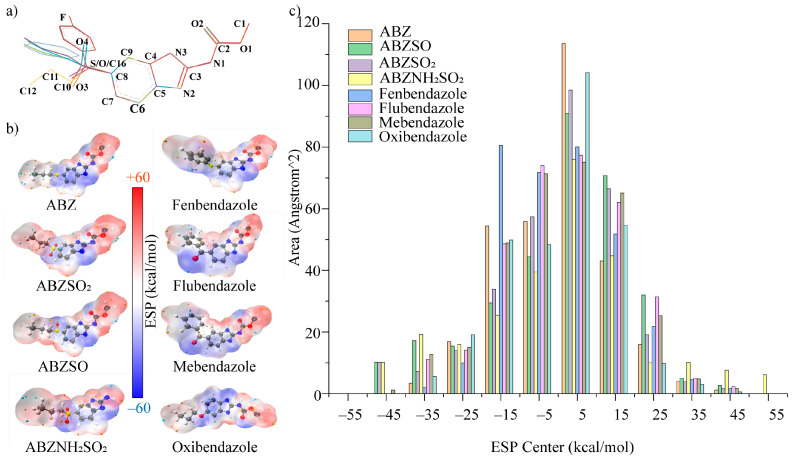
(**a**) The alignment of cross-reactive analytes with ABZ based on lowest energy conformations with all of the hydrogen atoms hidden. (**b**) Electrostatic potential energy of analytes. The negative potential areas are indicated in blue; red coloring indicates positive potential areas, and white indicates relatively neutral areas. The blue and golden globules on the surface represent the minima and maxima of ESP (kcal/mol) on the van der Waals surface. (**c**) The calculated surface area distribution in different ESP ranges on the van der Waals surface.

The hapten design of this work was based on the conjecture that the sulfanyl/sulfonyl moiety is the characteristic moiety of ABZs, thus should be retained to prepare ABZs antibodies. Figure 3a shows that when this moiety of the structure is changed, such as S atom (ABZ, in green) becomes O atom (oxibendazole, in yellow), chain alkyl group (ABZ, in green) becomes phenyl group (fenbendazole, in light blue), or both (flubendazole in brick-red, or mebendazole in grey), etc., at least a 10-fold decrease in CRs was observed, indicating the criticality of the sulfanyl/sulfonyl group of the structure. It can be seen in Figure 3a that all molecules are well coincided with ABZ, and the RMSD values of the alignments are from 2.3 × 10^−3^ of ABZNH_2_SO_2_ to 1.3 × 10^−2^ of ABZSO_2_ and fenbendazole (Table 3), demonstrating the extent of the difference of other molecules with ABZ, which agreed with CR data on the whole. It can also be seen in Figure 3a, the substitution of carbon or oxygen atom for sulfur atom in flubendazole (in red), mebendazole (in gray), or oxibendazole (in gray) causes changes in torsion angle of the left group towards the benzimidazole ring comparing with that of ABZ (in red), which might be partly responsible for the 10-fold decrease of Mab recognition. The ABZSO (in blue), ABZSO_2_ (in purple), and ABZNH_2_SO_2_ (in orange) show almost the same angle, but the difference between CRs is up to dozens of times. There shall be other factors which influence the recognition of antibody besides the contribution of molecular conformation.

ESP describes the potential energy of a proton placed at a point near the molecule. As different substituents may affect the electrostatic field, ESP analysis helps to visualize spatial regions of molecules and to analyze electrostatic interaction between antibody and antigen [30,33]. The ESP calculations displayed on van der Waals surfaces of global lowest energy conformation are shown in Figure 3b, positive potential energy is represented by red areas around the molecules, of which the maxima were marked as golden globule, and these areas are repulsive to a proton, negative potential energy is represented by blue areas on the molecules with the minima marked as light blue globule, and these areas are attractive to a proton. It can be seen that the presence of the oxygen atom causes more negative potential surface. With consideration of almost identical structure of ABZNH_2_SO_2_ (CR = 1.9%) and ABZSO_2_ (CR = 76.9%), except for the methoxyamide group on the right of molecule in the case of ABZSO_2_ and amino group in the case of ABZSO_2_NH_2_, less positive potential surface can be noticed in ABZNH_2_SO_2_, and the reduction of electronic effect associated with the lack of group might influence the recognition by Mab. It is clearly seen from Figure 3c that the ESP surface area distribution of molecules in different ESP ranges are similar on the whole but obviously varied locally. Cross-reactive analytes seem to have stronger electronic effect (positive or negative area) than ABZ, but do not generate better CRs. The results imply that the electrostatic potential energy might play a limited role in the antibody recognition or has been masked by other powerful factors.

Critical molecular descriptors were finally compared as shown in Table 3. Studies have shown that molecular parameters such as lipid-water partition coefficient (Log *P*, a widely used measure of hydrophobicity), dipole moments (μ, represents the polarity of molecule, the larger the polarity, the stronger the hydrophilicity), and topological polar surface area (TPSA) of analytes are important factors affecting recognition by antibodies [34,35,36], and hydrophobic force was believed to be the main driving force between small molecules and antibody [30,34]. In this study, Log *P* was found to be positively correlated with antibody affinity. The hydrophobicity decreases when the sulfur group in the molecule is replaced with a sulfone or a sulfoxide groups, resulting in a decrease in CR. Compared with ABZ, the hydrophobicity of ABZNH_2_SO_2_ greatly decreases, which is the lowest among all the analytes as well as the CR. Other compounds with strong hydrophobic groups do not show high CRs, which may be due to the co-working conformational factors. Some other physicochemical parameters that may influence the immunogenicity of haptens, including MW, SAs, and several hydrophobic parameters has also been calculated and summarized in Table 3, which shows no significant relationship with the recognition.

In general, hydrophobicity still dominates in the process of recognition in this study, and conformational factors play a partial role. Changes in the part of what we maintained as epitope in hapten design, the sulfanyl group, cause decrease in antibody recognition. This confirms the correctness of the hapten design strategy in this study, that is, the characteristic moiety of the target molecule should be maximally exposed.

### 3.4. Matrix Effect and Recovery in Samples

Milk, muscle, and liver samples from bovine were chosen to determine matrix effect and recovery. Direct dilution following sample extraction was used as a simple way to eliminate matrix effects for rapid screening purpose. The milk extracts were diluted 5/10/20 times with PBS and the tissue samples extracts were diluted 10/20/40 times with PBS. The standard curves of ABZs prepared in diluted samples were then compared with those in PBS to evaluate the matrix effects (Figure 2e–j).

Considering both A_max_ and IC_50_ performance, 10-fold dilution of milk samples and 20-fold dilution of tissue samples (beef and liver) in PBS were chosen to establish the calibration curves. The calculated LODs of ABZ, ABZSO_2_, ABZSO, and ABZNH_2_SO_2_ were at 0.05, 0.05, 0.05, and 0.49 μg/L in milk samples, respectively, and the LODs of ABZNH_2_SO_2_ in beef and bovine liver samples were 1.12 and 0.56 μg/kg, which were below the MRL of ABZs. Negative foodstuffs were spiked with ABZs at concentrations of 0.5/2.0/10.0 or 10/30/90 μg/kg (L), and the average absorbance values were interpolated with calibration curves to determine recoveries. The linearity ranges (from IC_20_ to IC_80_), recoveries, and CVs of ABZs in spiked milk samples are shown in Table 4, and that of ABZNH_2_SO_2_ in spiked tissue samples are shown in Table 5. As shown in Table 4 and Table 5, the recoveries ranged from 60% to 108.8%, with CVs less than 15.9%, which confirmed that the ic-ELISA performed well in various matrices.

## 4. Conclusions

In this study, a new hapten was designed by exposing the characteristic sulfanyl group of ABZ as an epitope. Furthermore, one Mab 12F12 for simultaneously detection of ABZs including ABZNH_2_SO_2_ was prepared for the first time and with the highest affinity to date. The established ic-ELISA based on the Mab 12F12 presented times lower IC_50_ values than previously reported and was suitable for the screening of ABZs in foodstuffs. The molecular recognition mechanism was briefly explained via computational chemistry analysis and indicated that the hydrophobicity of molecules and conformational factors might be the key factors that affect the binding between antibody and ABZs in this study, which might offer guides for antibody preparation to reduce the cost of trial-and-error in subsequent research.

## Figures and Tables

**Table 1:** Specificities of reported antibodies against ABZs in the literatures.

Compounds			Haptens&IC_50_ (μg/L)	
	[22]	[21]	[20]	This study
	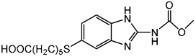	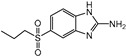	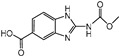	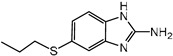
ABZ	1.4	>10,000	0.66	0.2
ABZSO_2_	1.8	1253.2	5.34	0.26
ABZSO	1.5	2241.4	2.91	0.77
ABZNH_2_SO_2_	>10,000	85.2	>1000	10.5
Carbendazim	- ^1^	-	14.84	>312.5
Fenbendazole	3.8	>10,000	0.75	1.68
Fenbendazole sulfone	8.3	-	6.27	-
Flubendazole	0.63	>10,000	0.37	3.68
Mebendazole	2.4	>10,000	0.3	4.14
Oxfendazole	0.62	>10,000	19.99	>312.5
Oxibendazole	1.4	>10,000	0.64	2.29
Parbendazole	-	-	1.13	-
Cambendazole	>100	-	>1000	-
Thiabendazole	>100	>10,000	-	>312.5

^1^ Not mentiond or not detected.

**Table 2:** Experimental IC_50_ values and cross-reactivities of ABZs in optimized ic-ELISA.

Analytes		IC_50_ (μg/L)	CR (%)
ABZ	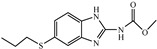	0.20	100
ABZSO2	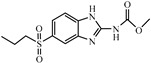	0.26	76.9
ABZSO	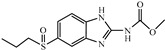	0.77	26.0
ABZNH2SO2	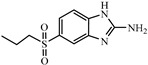	10.50	1.9
Fenbendazole	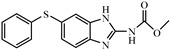	1.68	11.9
Flubendazole	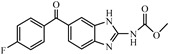	3.68	5.4
Mebendazole	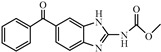	4.14	4.8
Oxibendazole	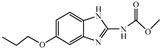	2.29	8.7
Oxfendazole	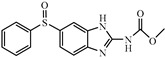	>312.5	<0.01
Triclabendazole	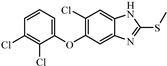	>312.5	<0.01
Carbendazim	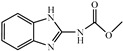	>312.5	<0.01
Thiabendazole	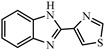	>312.5	<0.01

**Table 3:** Comparison of cross-reactivities and molecular descriptors of benzimidazoles.

Items	ABZ	ABZSO_2_	ABZSO	ABZNH_2_SO_2_	Fenbendazole	Flubendazole	Mebendazole	Oxibendazole
CR (%)	100	76.9	26.0	1.9	11.9	5.4	4.8	8.7
MW	265.1	297.3	281.3	239.3	299.3	313.3	295.3	249.3
volume(Å^3^)	320.9	336.4	328.5	201.8	255.3	261.1	256.2	225.0
Log *P*	2.8	1.2	1.2	1.0	3.4	3.1	2.9	2.4
μ	4.1	7.6	4.7	6.5	4.8	6.6	6.8	1.8
TPSA (Å^2^)	67.0	101.2	84.1	88.9	67.0	84.1	84.1	76.2
alignment RMSD	0	1.3 × 10^−^^2^	1.1 × 10^−^^2^	2.3 × 10^−^^3^	1.3 × 10^−^^2^	8.5 × 10^−^^3^	8.4 × 10^−^^3^	5.4 × 10^−^^3^

**Table 4:** Recoveries, CVs, LODs, and linearity range of ABZs in spiked milk samples using ic-ELISA. (*n* = 3).

	ABZs in Milk Samples				
	Spiked Level (μg/L)	Recovery (%)	CV (%)	LOD (μg/L)	Linearity Range (μg/L)
ABZ	0.5	87.5%	1.0%	0.05	0.08–0.4
	2	82.1%	7.7%		
	10	78.7%	6.9%		
ABZSO_2_	0.5	94.2%	4.1%	0.05	0.08–0.5
	2	71.4%	10.5%		
	10	60.0%	6.8%		
ABZSO	0.5	105.1%	11.9%	0.05	0.1–2.5
	2	98.7%	9.9%		
	10	74.5%	7.9%		
ABZNH_2_SO_2_	10	108.0%	10.3%	0.50	1.5–73.5
	30	76.6%	12.3%		
	90	75.2%	2.9%		

**Table 5:** Recoveries, CVs, LODs, and linearity range of ABZNH_2_SO_2_ in spiked tissue samples using ic-ELISA. (*n* = 3).

	ABZNH_2_SO_2_ in Tissue Samples				
	Spiked Level (μg/kg)	Recovery (%)	CV (%)	LOD (μg/kg)	Linearity Range (μg/kg)
Beef	10	84.1%	2.4%	1.12	2.3–25.9
	30	91.6%	4.9%		
	90	74.2%	6.6%		
Liver	10	87.8%	4.5%	0.56	1.3–23.7
	30	106.1%	9.1%		
	90	108.8%	15.9%		

## Data Availability

Not applicable.

## Supplementary Materials

The following are available online at https://www.mdpi.com/article/10.3390/foods10123106/s1, Figure S1: 1 H NMR spectra of hapten.
