# Supplementary material for: Hapten Synthesis and Monoclonal Antibody Preparation for Simultaneous Detection of Albendazole and Its Metabolites in Animal-Origin Food
Source: Foods. 2021 Dec 14;10(12):3106. doi: 10.3390/foods10123106 (PMC8700926; doi:10.3390/foods10123106)
Supplement: Supplementary file 1 [file foods-10-03106-s001.zip › foods-1478410-supplementary.pdf]

Supplementary Material

Buffers

The common buffer solutions used in the experiment are listed:

(1) Coating buffer (0.05 mol L−1 carbonate buffer, pH 9.6)

(2) Phosphate buffer solution (PBS buffer, 0.01 mol L^{-1}, pH 7.4)

(3) Blocking buffer (2% skim milk powder (w/v))

(4) Washing buffer (PBST, PBS buffer with 0.05% Tween–20 (v/v), pH 7.2)

(5) antibody diluention buffer (PBS buffer with 0.2% albumin, (w/v));

(6) Goat anti-mouse IgG (HRP labeled) dilution buffer (PBS buffer with 5% albumin (w/v))

(7) Stopping reagent (2 mol L^{-1} H_2SO_4)

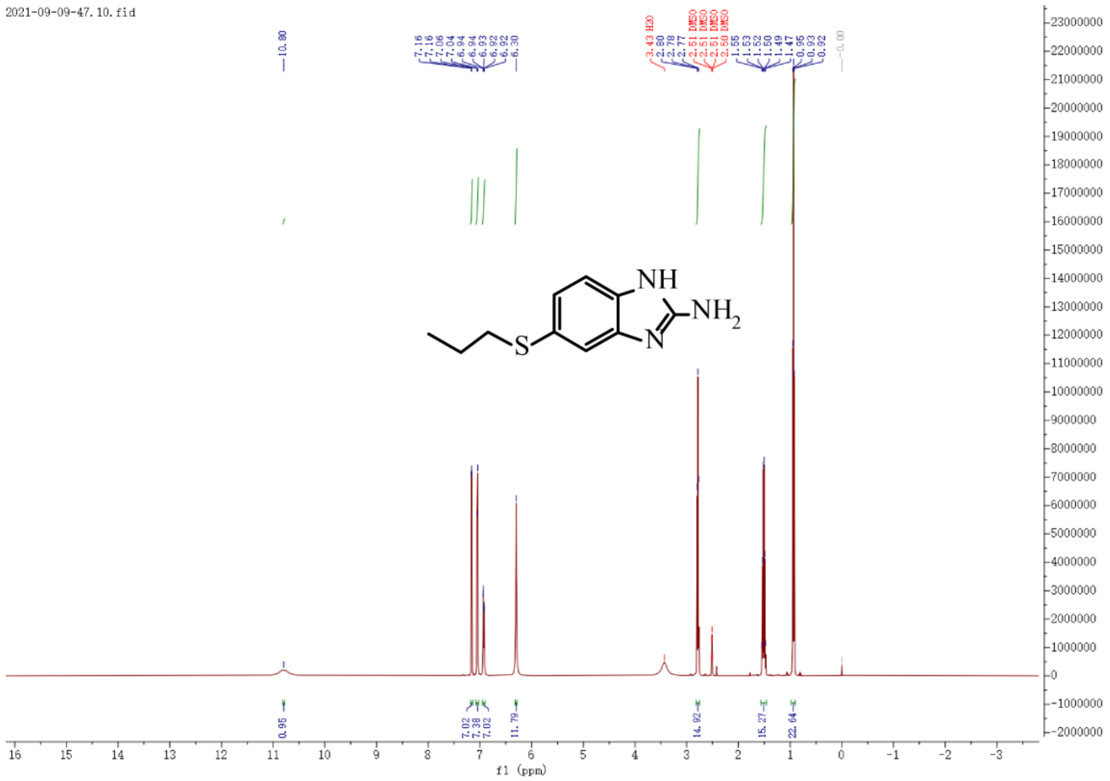

Figure S1. ^{1}H NMR spectra of hapten.
